# Surgical Site Infection Following Caesarean Section by *Acinetobacter* Species: A Report from a Hyperendemic Setting in the Brazilian Amazon Region

**DOI:** 10.3390/microorganisms9040743

**Published:** 2021-04-02

**Authors:** Blenda Gonçalves Cabral, Danielle Murici Brasiliense, Ismari Perini Furlaneto, Yan Corrêa Rodrigues, Karla Valéria Batista Lima

**Affiliations:** 1 Parasitic Biology in the Amazon Region, Center of Biological and Health Sciences, State University of Pará, Belém 66087-662, PA, Brazil; blendacabral.m@gmail.com (B.G.C.); yan.13@hotmail.com (Y.C.R.); karlalima@iec.gov.br (K.V.B.L.); 2 Bacteriology and Mycology Section, Evandro Chagas Institute, Ananindeua 67030-000, PA, Brazil; 3 Health Education, University Center of Pará, Belém 66613-903, PA, Brazil; ismaripf@hotmail.com

**Keywords:** caesarean, surgical site infection, *Acinetobacter*, clonal complexes

## Abstract

Surgical site infection (SSI) following caesarean section is associated with increased morbidity, mortality, and significant health care costs. This study evaluated the epidemiological, clinical, and microbiological features of *Acinetobacter* spp. in women with SSIs who have undergone caesarean section at a referral hospital in the Brazilian Amazon region. This study included 69 women with post-caesarean SSI by *Acinetobacter* spp. admitted to the hospital between January 2012 and May 2015. The 69 *Acinetobacter* isolates were subjected to molecular species identification, antimicrobial susceptibility testing, detection of carbapenemase-encoding genes, and genotyping. The main complications of post-caesarean SSI by *Acinetobacter* were inadequate and prolonged antibiotic therapy, sepsis, prolonged hospitalization, and re-suture procedures. *A. baumannii*, *A. nosocomialis* and *A. colistiniresistens* species were identified among the isolates. Carbapenem resistance was associated with OXA-23-producing *A. baumannii* isolates and IMP-1-producing *A. nosocomialis* isolate. Patients with multidrug-resistant *A. baumannii* infection showed worse clinical courses. Dissemination of persistent epidemic clones was observed, and the main clonal complexes (CC) for *A. baumannii* were CC231 and CC236 (Oxford scheme) and CC1 and CC15 (Pasteur scheme). This is the first report of a long-term *Acinetobacter* spp. outbreak in women who underwent caesarean section at a Brazilian hospital. This study demonstrates the impact of multidrug resistance on the clinical course of post-caesarean infections.

## 1. Introduction

Among surgical site infections (SSIs), caesarean section is considered an important risk factor for postpartum infections due to uterine skin rupture, bladder catheterization, and contact with health care workers (HCWs) [1,2]. SSI following caesarean section is associated with increased morbidity and mortality, prolonged hospitalization, secondary infertility, and increased health care costs [3]. However, caesarean section is one of the most commonly performed surgical procedures worldwide, with reported frequencies ranging from 15% to 60% and associated postpartum bacterial infection rates of up to 25%, which is approximately 5–20 times higher than those of vaginal delivery [4,5].

Risk factors associated with post-caesarean SSI are classified into intrinsic (age and body mass index) and extrinsic (limited prenatal consultations, smoking, diabetes mellitus, obesity, hypertensive disorders, and anemia) factors [6]. During the intrapartum stage, other risk factors are also relevant, including emergency caesarean section, prolonged labor, premature rupture of membranes, and chorioamnionitis [7]. Moreover, complications generated by post-caesarean SSI include prolonged healing time after surgery due to several factors, e.g., wound dehiscence, prolonged hospital admission, prolonged use of antibiotics, possibility of re-admission and re-suture, sepsis and, in rare cases, mortality [1].

Several bacterial agents have been associated with post-caesarean SSI. Among Gram-positive bacteria, *Staphylococcus aureus* was responsible for 20–30% of hospital SSI. Other frequently isolated organisms are Gram-negative bacilli, including *Pseudomonas aeruginosa, Klebsiella* spp., and *Escherichia coli* [8]. Despite rarely being isolated from SSI postpartum patients, *Acinetobacter* species are considered to have major clinical relevance, especially *A. baumannii,* which causes bacteraemia, pneumonia, skin and soft tissue infections, and meningitis [9]. The World Health Organization (WHO) has classified this species as a critical-tier priority pathogen for antimicrobial development owing to its high resistance rates [10,11]. Additionally, other *Acinetobacter* species, such as *A. nosocomialis* and *A. pittii*, have been increasingly associated with hospital-acquired infections (HAIs) [9]. Despite the phenotypic similarities, differences between species of *Acinetobacter* have been described, mainly in relation to virulence factors and susceptibility profiles to antimicrobials [12].

Several molecular typing methods are applied to the study of molecular epidemiology, clonal relatedness studies, and assessments of transmission of bacterial pathogens. Semi-automated repetitive extragenic palindromic polymerase chain reaction (rep-PCR) on the DiversiLab^®^ System, one of the most effective fingerprinting methods, has a discriminatory feature similar to pulsed-field gel electrophoresis (PFGE), the standard technique for *A. baumannii* typing [13]. Multilocus sequence typing (MLST) is a tool widely used for molecular epidemiological investigations, providing insights into the global population structure, genetic diversity, and high-risk clone identification [14].

The present study first describes the epidemiological, clinical, and microbiological aspects related with post-caesarean SSI at a hyperendemic setting for *Acinetobacter* spp. at a referral hospital in Belém, Amazon Region, Brazil.

## 2. Materials and Methods

### 2.1. Study Design and Clinical Data Collection

This was a single-center, retrospective cohort study, performed at a 484-bed tertiary-care teaching hospital for high-risk pregnant women and newborns, located in Belém, Amazon Region, Brazil. The present study included women who underwent caesarean delivery and were admitted to the obstetric ward and with a surgical wound culture positive for *Acinetobacter* spp., between January 2012 and May 2015. Demographic, epidemiological, and clinical data were obtained from patients’ medical records.

Surgical site infection was defined according to criteria designed by the Centers for Disease Control and Prevention [15]. This study was approved by the Institutional Ethics Committee of Evandro Chagas Institute (N° 2.999.939/05.Nov.2018) and the hospital under study (N° 3.126.011/30.Jan.2019). Written informed consent was obtained from all participants.

### 2.2. Bacterial Isolates and Antimicrobial Susceptibility Testing

*Acinetobacter* spp. isolates included in the study were obtained from surgical wound cultures of women with post-caesarean SSI. Preliminary identification was performed on the Vitek-2 System (bioMérieux, Marcy l’Etoile, France) in the hospital laboratory, and samples were sent to the Evandro Chagas Institute for further assays.

Antimicrobial susceptibility testing (AST) was performed on the Vitek-2 System (bioMérieux, Marcy l’Etoile, France), according to Clinical and Laboratory Standards Institute (CLSI) criteria [16]. Multidrug resistance (MDR) was defined as reduced or lack of susceptibility of an organism to three or more antimicrobial classes [17].

### 2.3. Acinetobacter Species Identification

Species identification was performed through *rpo*B partial sequencing, as described earlier [18]. Obtained sequences were compared to those available on the GenBank database using the BLAST search engine (http://blast.ncbi.nlm.nih.gov/Blast.cgi, accessed on 30 December 2020).

### 2.4. Detection of Carbapenemase-Encoding Genes

All isolates non-susceptible to imipenem and/or meropenem were investigated for the presence of carbapenem-hydrolyzing class D β-lactamase (CHDL) (*bla*_OXA-23-like_, *bla*_OXA-24/40-like_, *bla*_OXA-51-like_, *bla*_OXA-58-like_ and *bla*_OXA143-like_) and metallo-β-lactamase (*bla*_NDM-like_, *bla*_IMP-like_, *bla*_VIM-like_ and *bla*_SPM-like_)-encoding genes by polymerase chain reaction (PCR) using primers as previously described [19,20].

### 2.5. Genotyping by rep-PCR and Multilocus Sequence Typing

Genetic relatedness of the isolates was assessed using semi-automated rep-PCR on the DiversiLab^®^ System (bioMérieux) after following the manufacturer’s guidelines. Data were analyzed and interpreted using internet-based DiversiLab software (v. 3.4) with the Kullback-Leibler correlation coefficient. Strains presenting a similarity of ≥95% were considered clonally related [13]. Multilocus sequence typing (MLST) was performed on *A. baumannii* isolates that were not susceptible to carbapenems following protocols previously described by Bartual et al. 2005 [21] and Diancourt et al. 2010 [22] available at the PubMLST website (http://pubmlst.org/acinetobacter, accessed on 30 December 2020).

### 2.6. Statistical Analysis

Data were expressed as absolute and relative (percentage) frequencies for categorical variables or mean ± standard deviation (SD) for continuous variables. Non-parametric tests such as Fisher’s exact test and the G-test of independence were performed using the program BioEstat version 5.5 or GraphPad Prism version 8.00. *p* values ≤ 0.05 were considered indicative of statistical significance.

## 3. Results

### 3.1. Demographic and Clinical Data

From January 2012 to May 2015, a total of 69 women submitted to caesarean section at the study hospital acquired a surgical site infection by *Acinetobacter* spp. The mean age of the women was 25 years (12 to 46 years, SD: 7 years) and 100% of caesarean sections were emergency procedures. Clinical and sociodemographic data are presented in Table 1. During the gestational period, 51% (*n* = 35) of women had gynecological complications such as a urinary tract infection (UTI) and leukorrhea. Additionally, 19% (*n* = 13) of women had previously suffered between one and three miscarriages. Post-caesarean section infection by *Acinetobacter* spp. was more frequent in primiparous women (49% of cases, *n* = 34).

The main comorbidities in the clinical histories of the 69 women were systemic arterial hypertension (14%) and asthma (4%). Underlying diseases observed in the family history of patients included hypertension (26%; *n* = 18) and diabetes mellitus (22%; *n* = 15). Of the 69 women, nine (13%) were smokers and 13 (19%) alcoholics.

SSI was detected, on average, on the tenth day (interquartile range: 7 and 14 days) after the caesarean section procedure, and in 45% (*n* = 31) of the cases, the patients were readmitted to the hospital due to symptoms of post-caesarean infection.

### 3.2. Confirmation of Acinetobacter Species, Antimicrobial Susceptibility Testing, and Carbapenemase Gene Detection

Molecular identification of *Acinetobacter* spp. revealed three species associated with SSI in the 69 women: *A. baumannii* (71%, *n* = 49), *A. nosocomialis* (28%, *n* = 19), and *A. colistiniresistens* (1%, *n* = 1). Among the isolates of *A. baumannii,* 61% (30/49) showed resistance to carbapenems and were positive for the *bla*_OXA-23-like_ and *bla*_OXA-51-like_ genes; these isolates were also resistant to all β-lactams tested, ciprofloxacin, and gentamicin. Amikacin, tigecycline, and colistin were the most effective antimicrobial agents against carbapenem-resistant *A. baumannii* in vitro (Table 2). Significant differences in antimicrobial resistance rates were observed between *A. baumannii* and *A.* non-*baumannii* isolates (Table 2). Among *A.* non-*baumannii* isolates, only one *A. nosocomialis* isolate was resistant to carbapenems and harbored the *bla*_IMP-1_ gene. This isolate showed high minimal inhibitory concentration (MIC) values in response to the β-lactams tested but was susceptible to ciprofloxacin, aminoglycosides, tigecycline and colistin. *A. colistiniresistens*, showed resistance to colistin, an intrinsic characteristic of the species, and susceptibility to all other tested antimicrobials.

### 3.3. Complications of Post-Caesarean SSI by Susceptible and Carbapenem-Resistant Acinetobacter spp.

To assess the impact of carbapenem resistance in the clinical evolution of cases, patients were divided into two groups: SSI by (I) carbapenem-susceptible *Acinetobacter* and (II) carbapenem-resistant *Acinetobacter*. Patients in the carbapenem-resistant group (*n* = 31) demonstrated a more significant association with complications and prolonged post-caesarean SSI evolution than the carbapenem-susceptible group (*n* = 38) (Table 3). Post-caesarean SSI due to carbapenem-resistant *Acinetobacter* was also associated with a prolonged hospital stay (6 to 80 days) and an average discharge of 28 days.

It is worth noting that 93% (64/69) of all women with post-caesarean SSI by *Acinetobacter* were submitted to at least one re-suture procedure. In addition, 16 (23%) underwent other surgical procedures, due to the severity of the infection, including total hysterectomy (*n* = 4), exploratory laparotomy (*n* = 4), debridement (*n* = 2), hematoma drainage (*n* = 6), vessel ligation (*n* = 2), and graft insertion (*n* = 1). Four cases of SSI by carbapenem-resistant *A. baumannii* evolved to septicemia and three of these patients underwent other surgical procedures, such as exploratory laparotomy and total hysterectomy with a longer duration of hospital stay, ranging from 39 to 80 days (mean 32 days, SD: 16 days).

In relation to the antimicrobial therapy used for the treatment of SSI by *Acinetobacter*, the most used empirical therapies were the combination of penicillin, gentamicin, and metronidazole (39%), followed by ceftriaxone (23%) and oxacillin (16%). For carbapenem-resistant isolates in 94% of cases, the empirical therapy was inadequate. After the antimicrobial susceptibility testing results (adjusted therapy), the most used antimicrobials were ciprofloxacin (21.7%), amikacin (14.5%), and polymyxin B (5.8%).

### 3.4. rep-PCR and MLST Genotyping

All *A. baumannii* (*n* = 49) and *A. nosocomialis* (*n* = 19) isolates were genotyped by rep-PCR. *A. baumannii* isolates revealed 29 distinct genetic patterns (P), of which nine clusters were genetically related isolates (similarity > 95%) and 20 had unique profiles.

**Figure 1:**
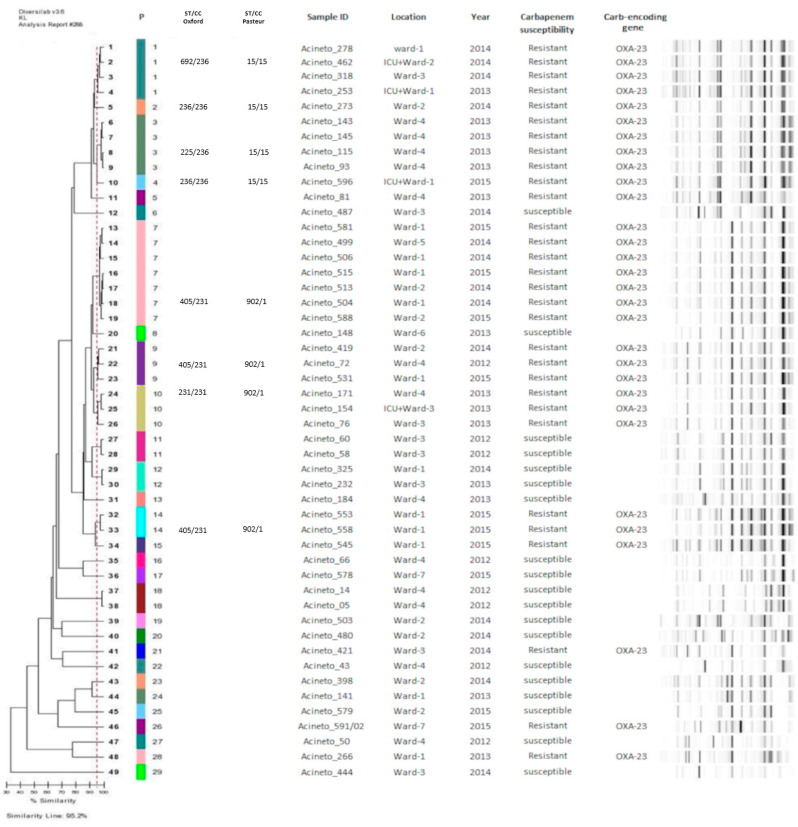
Dendrogram of *A. baumannii* isolates analysed in the study. Abbreviations: P: Patterns (≥95% similarity), ST: sequence type, CC: clonal complexes. Sample ID: Identification of *A. baumannii* isolates obtained from surgical site of caesarean section patients. Location: Obstetric wards in which women remained after caesarean section surgery.

Among the nine clusters, the most prevalent were P7 (seven isolates), P3 (five isolates), and P1 (four isolates). The P7 clonal group was formed by OXA-23-producing *A. baumannii* and was previously detected at the hospital in 2014 and 2015, circulating in three different obstetric wards and was associated with ST405/CC231. In addition, P1, P9, P10 clonal groups of OXA-23-producing *A. baumannii* also showed dissemination between different obstetric wards (Figure 1).

**Figure 2:**
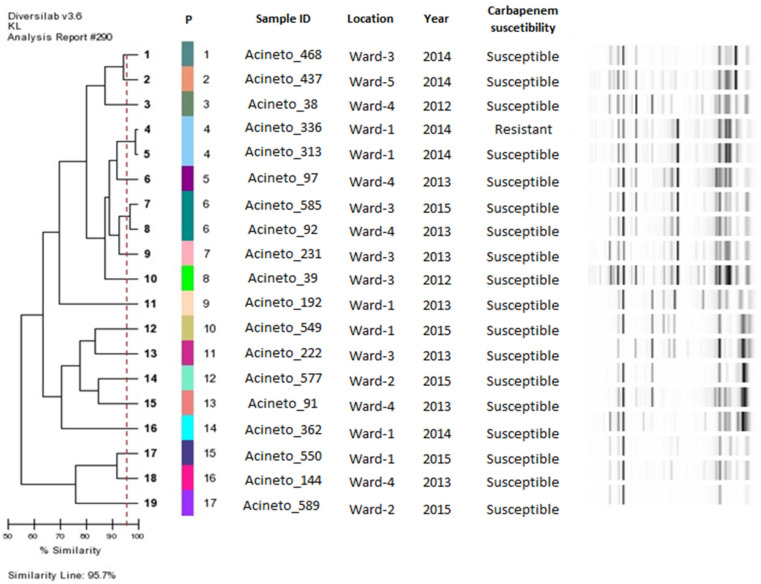
Dendrogram of *A. nosocomialis* isolates analysed in the study. Abbreviations: P: Patterns (number of genetic patterns identified by DiversiLab System analysis, formed with ≥95% similarity). Sample ID: Identification of *A. nosocomialis* isolates obtained from surgical site of caesarean section patients. Location: Obstetric wards in which women remained after caesarean section surgery.

A wide diversity of clonal patterns was observed among the 19 isolates of *A. nosocomialis*. Seventeen distinct genetic patterns were identified, among which two clusters of two isolates each existed. Clonal dissemination between the different wards was identified only in the P6 group (Figure 2).

Analysis of representative isolates from different clusters of *A. baumannii* by MLST revealed that the isolates belonged to epidemic clones, including the ST225, ST236, ST692 (CC236); ST231, and ST405 (CC231), according to the Oxford scheme, and ST902 (CC1) and ST15 (CC15), according to the Pasteur scheme (Figure 1).

## 4. Discussion

SSI is one of the most common complications after caesarean delivery, occurring in approximately 10% of cases. Post-caesarean section SSI is a major cause of prolonged hospital stays and increasing healthcare costs. In Brazil, more than 1 million caesarean deliveries are performed annually, with SSI rates of 1.42% (2018). However, this number likely reflects underreported cases, since this type of infection only became mandatory to report in 2014 [23].

The most commonly reported pathogens in post-caesarean SSI include *Staphylococcus aureus*, *Streptococcus* spp., and members of the Enterobacteriaceae family [24]. *Acinetobacter* spp. are not commonly reported as a causative agent post-caesarean SSI. Herein, we report the occurrence of 69 cases of post-caesarean SSI related to three distinct species of *Acinetobacter* and the impact of multidrug resistance on the clinical evolution of patients.

All patients who developed SSI of *Acinetobacter* spp. underwent an emergency caesarean section procedure, which is a known risk factor for developing SSI [5]. The main indications for performing caesarean section in our study were fetal suffering, premature rupture of membranes, and hypertensive diseases of pregnancy. This was expected because the hospital under study is a reference hospital for high-risk pregnancies. It is also known that premature rupture to the membrane contributes to the colonization of the microbiota from the lower genital tract in the amniotic fluid, leading to wound and peritoneal cavity contamination [25].

Diagnosis of a UTI during pregnancy is another important risk factor for SSI, which was observed in 51% (*n* = 35) of women in this study. Women diagnosed with UTIs during pregnancy are three times more likely to develop post-caesarean SSI [26]. Therefore, strict surveillance measures must be taken during prenatal care to diagnose UTIs and offer treatment.

Post-caesarean infections have been reported to increase maternal morbidity and mortality. Puerperal sepsis is the third most common cause of maternal mortality, accounting for 10.7% of maternal deaths worldwide [26]. In our study, 13% of patients with SSI evolved to septicaemia and were transferred from the obstetric ward to the ICU due to clinical complications. However, no deaths were observed. All cases of sepsis occurred in patients with multidrug-resistant *A. baumannii* infection.

The cases of SSI occurred, on average, 10 days after the caesarean section. In 45% of cases, the patients were readmitted with signs and symptoms of infection in the surgical wound, a fact that may be associated with the lack post-discharge surveillance. Since SSI may take several days to become apparent and the average length of the post-operative stay in the hospital following caesarean delivery has gradually reduced to three days or less, methods that assure active post-discharge surveillance are an important prerequisite for the effective surveillance of SSI following caesarean delivery [27].

The spread of broad antimicrobial resistance strains of *Acinetobacter* is posing a challenge to infections treatment, leading to high morbidity, mortality, and longer hospital stays. This scenario of multidrug-resistance has also led to an increase in the antimicrobial therapy based on the combination of two of more antibiotics for treatment of multidrug-resistant *A. baumannii* infections [11]. In the present study, most of the patients received prolonged and inadequate antibiotic therapy, which was associated with carbapenem-resistant isolates. In addition, both empirical and adjusted antibiotic therapy was based on the traditional strategy of combining antibiotics from different classes; however, only after antimicrobial susceptibility testing of the correct antibiotic was therapy applied, which was mainly based on ciprofloxacin, amikacin, and polymyxin B. Finally, our data demonstrates the importance of AST and encouraging teams of professionals to gain prior knowledge of hospital bacterial resistance when prescribing antibiotics for treatment and prophylaxis of SSI [28,29].

This study demonstrated an association between septicemia and other complications, such as the need for further surgery, extended hospital stay (6 to 80 days), and OXA-23-producing *A. baumannii* infection, with the epidemic clonal complexes CC236 (ST692, ST225 and ST236) and CC231 (ST231 and ST405) (Oxford scheme), and ST902 (CC1) and ST15 (CC15) (Pasteur scheme), demonstrating the severity of these specific infections. In Brazil, the high levels of carbapenem-resistance have been associated with the spread of OXA-23-producing *A. baumannii* strains, particularly those included in the clonal complexes CC 104, CC 109, and CC113 (Oxford scheme) [30]. Although not among the most prevalent, epidemic CC15 and CC1 (Pasteur scheme) have been reported in other Brazilian hospitals to be associated with cases of bloodstream infection and meningitis [31]. Moreover, CC15 (Pasteur scheme) was the second most prevalent complex in Buenos Aires and Rosario, Argentina, in a study conducted by Stietz and collaborators [32]. Finally, genotyping revealed the permanence of different epidemic clones of *A. baumannii* over the years of the study as well as their spread between different obstetric wards of the hospital, highlighting the ability of these clones to spread and remain in the hospital over time.

Despite previous reports linking *A. nosocomialis* isolates to susceptibility to several antimicrobial classes, this species has emerged as an important pathogen worldwide owing to its increasing prevalence in nosocomial infections and capacity to acquire various mechanisms of antimicrobial resistance [33]. In our study, 28% of SSI cases were related to this species. We emphasize that these isolates were mistakenly identified as *A. baumannii* at the hospital using a semi-automated identification method, reinforcing the need for accurate species identification of *Acinetobacter* genus. An IMP-1-producing *A. nosocomialis* strain was identified, and to the best of our knowledge, this is the first report of this pathogen in a post-caesarean section SSI. This reinforces the clinical relevance of this emerging pathogen and highlights the need for further studies on virulence factors related to this species and others of the so-called *Acinetobacter baumannii*-*A. calcoaceticus* complex. One *A. colistiniresistens* strain was detected among our population. This species is rarely associated with HAIs and is associated with the peculiar feature of intrinsic resistance to colistin [34].

## 5. Conclusions

In conclusion, the present study firstly describes the clinical, epidemiological, and microbiological aspects related to post-caesarean SSI at a hyperendemic setting for *Acinetobacter* spp. in the Brazilian Amazon Region. Patients with carbapenem-resistant *A. baumannii* infection were associated with more complications, prolonged clinical evolution, and epidemic clonal lineages. Therefore, the implementation of effective infection control measures during caesarean section surgery and active post-discharge surveillance is essential in order to provide a safe postpartum experience. These measures will effectively reduce healthcare costs and help to alleviate the emotional and physical burden that caesarean section SSIs cause.

## Figures and Tables

**Table 1:** Patient characteristics and risk factors for developing post-caesarean section surgical site infection by *Acinetobacter* spp.

	*N*	%
**Gestational age (weeks)**		
Preterm (<37 weeks)	25	36%
Full-term (37 to 41 weeks)	36	52%
Post-term (>41 weeks)	0	0%
N.I	8	12%
**Personal morbidities**		
Chronic noncommunicable diseases	16	23%
Chronic communicable diseases	3	4%
Infectious diseases	4	6%
Anemia	3	4%
No morbidities	42	60%
N.I	2	3%
**Caesarean indication**		
CPD	4	6%
Hypertensive diseases of pregnancy	14	20%
PROM	7	10%
Fetal distress	14	20%
Fetal death	2	3%
Sexually Transmitted Infections	5	7%
Others	19	28%
N.I	4	6%
**Sexually transmitted infections**		
Yes	10	14%
No	52	75%
N.I	7	10%
**Abortion**		
Yes	13	19%
No	55	80%
N.I	1	1%
**Gynecological complications**		
Yes	35	51%
No	28	41%
N.I	6	9%
**Age (years)**		
12 a 19	20	29%
20 a 35	45	65%
36 a 46	4	6%
**Marital status**		
Married	6	9%
Single/unmarried	12	17%
Stable union	37	54%
N.I	14	20%
**Education level**		
Elementary and middle school	27	40%
High school	27	39%
University Education	3	4%
N.I	12	17%
**Profession/occupation**		
Housewife	30	43%
Student	8	12%
Teacher	2	3%
Autonomous	2	3%
Others	14	20%
N.I	13	19%

Abbreviations: CPD: Cephalopelvic disproportion, PROM: Premature rupture of membranes. Others: Amniotic Fluid Index, Restriction of Intrauterine Growth, Premature placental dislocation. Noncommunicable chronic diseases: systemic arterial hypertension, diabetes mellitus, asthma, epilepsy. Chronic communicable diseases: hepatitis B. Infectious diseases: malaria, leishmaniasis, pneumonia. N.I: No information.

**Table 2:** Antimicrobial susceptibility profile for *A. baumannii* and non-*A. baumannii* groups.

Antimicrobial	*A. baumannii* (N = 49)		Non-*A. baumannii* (N = 20)		*p*-Value *
	S	NS	S	NS	
SAM	18	31	19	1	<0.0001 ^†^
TZP	17	32	17	3	0.0002 ^†^
CTX	1	48	3	17	0.0702
CAZ	15	34	19	1	<0.0001 ^†^
FEP	15	34	19	1	<0.0001 ^†^
IMP	19	30	19	1	<0.0001 ^†^
MEM	19	30	19	1	<0.0001 ^†^
GEN	17	32	20	0	<0.0001 ^†^
AMK	33	16	20	0	0.0033 ^†^
CIP	15	34	20	0	<0.0001 ^†^
TIG	49	0	20	0	>0.9999
COL	48	1	20	0	>0.9999

* Fisher Exact test. ^†^ Statistically significant. Abbreviations: S: susceptible; NS: non-susceptible; SAM: ampicillin-sulbactam; TZP: piperacillin/tazobactam; CTX: cefotaxime; CAZ: ceftazidime; FEP: cefepime; IMP: imipenem; MEM: meropenem; GEN: gentamicin; AMK: amikacin; CIP: ciprofloxacin; TIG: tigecycline; COL: colistin.

**Table 3:** Clinical complications developed by women with post-caesarean surgical site infection by carbapenem-susceptible or -resistant *Acinetobacter* spp.

Complications	Carbapenem Resistant (N = 31) *n* (%)	Carbapenem Susceptible (N = 38) *n* (%)	*p*-Value *
Re-suture time (hours)			
>1	2 (6%)	2 (5%)	1.0000
<1	25 (81%)	30 (79%)	
Number of re-sutures			
1	17 (55%)	33 ^a^ (87%)	0.0007 ^†^
2	12 ^a^ (39%)	2 (5%)	
Other surgeries			
Yes	12 ^a^ (39%)	4 (11%)	0.0091 ^†^
No	19 (61%)	34 ^a^ (89%)	
Prolonged wound healing (days)			
1–20	9 ^b^ (29%)	25 ^a^ (66%)	0.0044 ^†^
21–40	18 ^a^ (58%)	10 ^b^ (26%)	
41–62	2 (6%)	0	
Culture (days)			
4–20	15 (48%)	32 ^a^ (84%)	0.0003 ^†^
21–40	9 ^a^ (29%)	2 ^b^ (5%)	
41–62	5 ^a^ (16%)	0 ^b^	
Sepsis			
Yes	4 ^a^ (13%)	0	0.0364 ^†^
No	27 (87%)	38 ^a^ (100%)	
Wound dehiscence			
Yes	23 (74%)	30 (79%)	0.7760
Not	8 (26%)	8 (21%)	
Hospitalization (days)			
6–30	13 (42%)	29 ^a^ (76%)	0.0060 ^†^
>30	18 ^a^ (58%)	9 (24%)	
Antibiotic use (days)			
3–11	0 ^b^	8 ^a^ (21%)	0.0003 ^†^
12–36	21 (68%)	28 (74%)	
>	10 ^a^ (32%)	2 ^b^ (5%)	
Antibiotic therapy			
Adequate	2 (6%)	35 ^a^ (92%)	<0.0001 ^†^
Inadequate	29 ^a^ (94%)	3 (8%)	
Nº of antimicrobials used in the therapy			
1–3	8 ^b^ (25.8%)	16 (42.1%)	
4–6	19 (61.3%)	22 (57.9%)	0.0271 ^†^
>6	4 (12.9%)	0 ^b^	

* Fisher’s Exact Test or G-Test of Independence (Chi-square Residue Analysis), as appropriate. ^†^ Statistically significant. ^a^ Frequency higher than expected at random. ^b^ Frequency lower than expected at random.

## Data Availability

All relevant data is presented in the present article.
